# Virtual biopsy using CT radiomics for evaluation of disagreement in pathology between endoscopic biopsy and postoperative specimens in patients with gastric cancer: a dual-energy CT generalizability study

**DOI:** 10.1186/s13244-023-01459-w

**Published:** 2023-07-05

**Authors:** Yiyang Liu, Shuai Zhao, Zixin Wu, Hejun Liang, Xingzhi Chen, Chencui Huang, Hao Lu, Mengchen Yuan, Xiaonan Xue, Chenglong Luo, Chenchen Liu, Jianbo Gao

**Affiliations:** 1 Department of Radiology, The First Affiliated Hospital of Zhengzhou University, Zhengzhou, 450052 China; 2 Henan Key Laboratory of Imaging Diagnosis and Treatment for Digestive System Tumor, Zhengzhou, 450052 China; 3 Department of Urology Surgery, The First Affiliated Hospital of Zhengzhou University, Zhengzhou, 450052 China; 4 Department of Gastroenterology, Xuanwu Hospital Capital Medical University, Beijing, 100053 China; 5 Department of Research Collaboration, R&D Center, Beijing Deepwise and League of PHD Technology Co., Ltd, Beijing, 100080 China; 6 Department of Gastroenterology, The Third Affiliated Hospital of Xinxiang Medical University, Xinxiang, 453003 China

**Keywords:** Single-energy CT imaging, Dual-energy CT imaging, Gastric cancer, Radiomics

## Abstract

**Purpose:**

To develop a noninvasive radiomics-based nomogram for identification of disagreement in pathology between endoscopic biopsy and postoperative specimens in gastric cancer (GC).

**Materials and methods:**

This observational study recruited 181 GC patients who underwent pre-treatment computed tomography (CT) and divided them into a training set (*n* = 112, single-energy CT, SECT), a test set (*n* = 29, single-energy CT, SECT) and a validation cohort (*n* = 40, dual-energy CT, DECT). Radiomics signatures (RS) based on five machine learning algorithms were constructed from the venous-phase CT images. AUC and DeLong test were used to evaluate and compare the performance of the RS. We assessed the dual-energy generalization ability of the best RS. An individualized nomogram combined the best RS and clinical variables was developed, and its discrimination, calibration, and clinical usefulness were determined.

**Results:**

RS obtained with support vector machine (SVM) showed promising predictive capability with AUC of 0.91 and 0.83 in the training and test sets, respectively. The AUC of the best RS in the DECT validation cohort (AUC, 0.71) was significantly lower than that of the training set (Delong test, *p* = 0.035). The clinical-radiomic nomogram accurately predicted pathologic disagreement in the training and test sets, fitting well in the calibration curves. Decision curve analysis confirmed the clinical usefulness of the nomogram.

**Conclusion:**

CT-based radiomics nomogram showed potential as a clinical aid for predicting pathologic disagreement status between biopsy samples and resected specimens in GC. When practicability and stability are considered, the SECT-based radiomics model is not recommended for DECT generalization.

**Critical relevance statement:**

Radiomics can identify disagreement in pathology between endoscopic biopsy and postoperative specimen.

**Graphical abstract:**

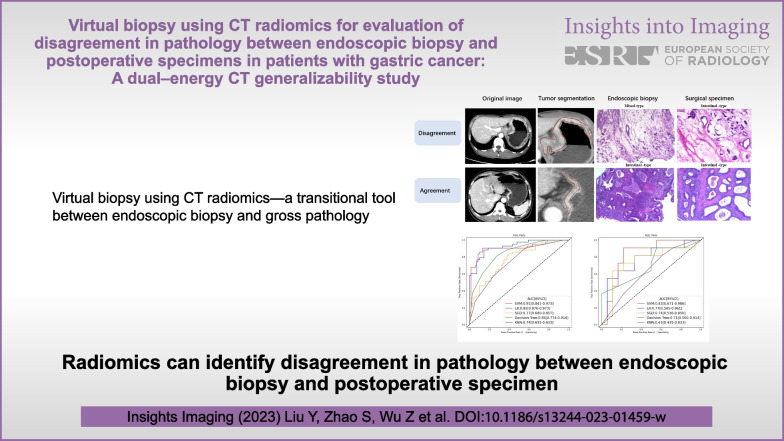

**Supplementary Information:**

The online version contains supplementary material available at 10.1186/s13244-023-01459-w.

## Key points

- Radiomics can identify disagreement in pathology between endoscopic biopsy and postoperative specimen.
- Multiple machine learning algorithms were applied to construct radiomics models.
- The SECT-based pathology-specific radiomics signatures cannot be stably generalized to DECT.

**Supplementary Information:**

The online version contains supplementary material available at 10.1186/s13244-023-01459-w.

## Introduction

Gastric cancer (GC) is the third leading cause of cancer-related mortality globally owing to its high heterogeneity [1]. Endoscopic biopsy is the current standard approach for GC diagnosis, which guides clinicians' decisions regarding the implementation of subsequent treatment for patients with GC. Reliable biopsy results can avoid unnecessary surgery and considerably decrease health care costs. However, endoscopy only captures limited tumor information from a small portion of the tumor tissue, providing an incomplete characterization of the tumor; therefore, biopsy results sometimes deviate from those of postoperative macroscopic pathology specimens [2]. In addition, studies have shown that the increasing popularity of endoscopy has increased the workload of pathologists and affected the accuracy of cancer diagnosis [3, 4]. Owing to these shortcomings, transitional or complementary tools to differentiate between endoscopic biopsy and postoperative specimen diagnosis are warranted.

Radiomics-enabled imaging biomarkers provide insights into the properties of the tumor phenotype that are imperceptible to human eyes and correlate intratumor heterogeneity with clinical outcomes [5, 6]. Therefore, the use of radiomics in cancer has expanded in recent years, triggering many research projects worldwide. Currently, the relationship between biopsy and postoperative specimens has been investigated for GC [7], rectal lesions [8], prostate cancer [9], spinal lesions [10], and skin carcinoma [11]. However, no study has focused on evaluating the pathologic disagreement between the two specimens in GC using a radiomics approach.

In medical imaging, it has been proved that dual-energy computed tomography (DECT) is superior to single-energy CT (SECT) because its spectral information contains unique attenuation properties that improve the visualization of biological processes and tissue characterization [12, 13]. Radiomics has also been successfully applied to the analysis of DECT images and has shown promising diagnostic and prognostic power in tumor research [14–16]. Brendlin et al. revealed that DECT radiomics approaches yield a remarkable additive value over SECT radiomics analysis in noninvasively predicting immunotherapy response in patients with stage IV melanoma [12]. Nevertheless, few attempts have been made to apply radiomics models trained on SECT to the validation cohort of DECT, which probably hinders radiomics generalizability in the field of CT imaging.

In this study, we aimed to develop and test SECT-based radiomics nomogram to identify the disagreement frequency between pathologic assessment based on an endoscopic biopsy and a postoperative specimen in GC patients, and to compare the performance of different machine learning algorithms for building radiomics signatures (RS). We also explored the feasibility of generalizing RS trained on SECT cohort to the DECT cohort. We hypothesize that RS based on SECT images at 120 kVp allows validation on 70 keV virtual monochromatic images (VMIs) derived from DECT, given the fact that they have similar photon energy levels and visual equivalence.

## Materials and methods

### Study population

This single-center observational study involved a retrospective single-energy imaging study for the training and testing of predictive models and a prospective dual-energy imaging validation study to assess the generalizability of the model.

Cohort 1 included patients who underwent SECT between May 2020 and May 2021. Cohort 2 prospectively recruited eligible patients who underwent DECT examination from March 2022 to September 2022. A total of 181 patients were recruited for the present study in accordance with the enrollment criteria (Additional file 1: Appendix. E1). Patients in the retrospective cohort were randomly divided into a training set (*n* = 112) and a test set (*n* = 29) at a ratio of 8:2, whereas all patients in the prospective cohort were included in the validation cohort (*n* = 40). The clinical data, enhanced pretreatment CT images, pathological results of endoscopic biopsies prior to treatment, and gross specimens after gastrectomy of patients with advanced GC who were treated with radical operations at our institutions were collected.

**Fig. 1:**
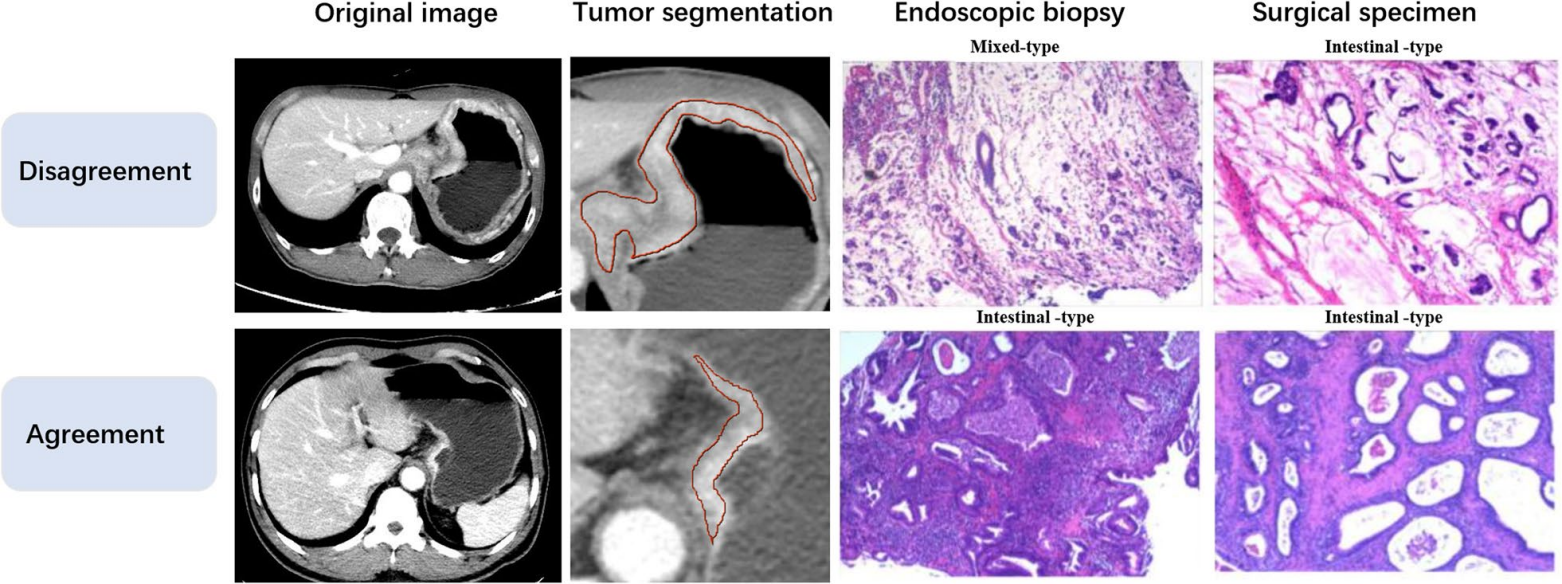
Typical original CT images, tumor segmentation, endoscopic biopsy and postoperative pathological images of two patients in pathologic disagreement and agreement group

All patients underwent endoscopy and gastrectomy for GC. Both endoscopic biopsy specimens and gross specimens resected from the primary site after surgery were pathologically analyzed to determine the Lauren classification using hematoxylin–eosin staining. Those with consistent Lauren classification of endoscopic biopsies and postoperative specimens were classified into the pathologic agreement group, and those with different Lauren classifications were classified into the disagreement group (Fig. 1). This study was approved by the Institutional Ethics Review Board, and the need for informed consent was waived owing to the observational design.

### Image acquisition and reconstruction parameters

All patients underwent abdominal dual-phase (arterial and venous)-enhanced CT after preparation for examination. Detailed patient preparation, imaging scheme, and acquisition parameters are summarized in Additional file 1: Appendix E2 & Table S1.

The dual-energy imaging-specific monoenergetic image subtypes were reconstructed at 70 keV energy level using a commercially available workstation (Advantage workstation, Version 4.7, GE Healthcare).

### Tumor segmentation and extraction of radiomics features

All CT DICOM images of the venous phase were transferred to prototypical software (Syngo Frontier, Radiomics 1.3.0, Siemens Healthineers, Germany) for tumor segmentation and radiomics feature extraction. A radiologist with 10 years of experience in gastrointestinal radiology performed the tumor segmentation. The details are provided in Additional file 1: Appendix E3. The intraclass correlation coefficients (ICCs) were calculated to assess reproducibility of the extracted radiomic features, and only those features with an ICC greater than 0.75 were retained for further analysis.

### Radiomic feature selection and radiomics signature building

All extracted radiomic features from each patient were normalized using the z-score method. Feature selection was carried out according to the following approach: Hierarchical analysis was performed based on Pearson’s correlation analysis, and the redundancy with correlation coefficients > 0.90 was eliminated. Subsequently, we performed feature selection using an analysis of variance (ANOVA) F-test statistic to select the top 30% features ranked by *F*-value (each feature has individual F-values related to target events).

Based on the final selection of features from the training set, five RS were constructed using different machine learning classifiers: Logistic Regression (LR), Support Vector Machine (SVM), Decision Tree (DT), Stochastic Gradient Descent (SGD), and K-Nearest Neighbors (KNN). The grid search method was used to select the optimal combination of hyperparameter values during training of each classification model.

### Best radiomics signature evaluation and its DECT generalization testing

The discriminative power of the five radiomics signatures obtained using the area under the receiver operating characteristic curve (AUC) and the 95% confidence intervals (CI) were computed. Delong test was used to evaluate the statistically significant differences in AUC values: (1) between five RS; (2) each RS in different cohorts. The best RS was defined as having the highest AUC value with no difference between the training and test sets, such RS had excellent discrimination and stability.

For cohort 1, the diagnostic value of the best RS was evaluated for all patients and subgroups defined by clinicopathological factors. Associations between the best RS and pathologic disagreement were assessed using Mann–Whitney U test analysis. Additionally, the performance of the best RS was validated in the DECT cohort to test its dual-energy generalization ability.

### Development and validation of the clinical-radiomics nomogram

Univariate analysis was used to assess the clinical variables for discriminating pathologic disagreement status in the training set, and those significant variables (*p* < 0.05) were included in multivariate logistic regression analysis to determine independent clinical predictors of disagreement status. A predictive model was developed by integrating the selected clinical predictor and the best RS using multivariable logistic regression. Moreover, the predictive model was visualized as a clinically applicable individualized nomogram. The discriminative power of the nomogram was assessed by using AUC value. Decision curve analysis (DCA) was performed to estimate the clinical usefulness of the nomogram by quantifying the net benefits at different threshold probabilities. To quantify the calibration of the nomograms, calibration curves accompanied by the Hosmer–Lemeshow test were plotted.

### Statistical analysis

Independent *t *test or the Wilcoxon rank-sum test was used to compare continuous variables between the two groups, while the chi-squared or the Fisher’s exact test was used to compare categorical variables. The AUCs, sensitivity, specificity, and positive and negative predictive values (PPV&NPV) were computed by using ROC curve analysis. AUC range 0.6–0.7 was considered as an indication of poor classification accuracy, 0.7–0.8 as fair, 0.8–0.9 as good, and 0.9–1.0 as excellent classification accuracy. The optimal cutoff point in the ROC curve was determined using Youden’s index. Moreover, the F1 score was computed for each model because of the imbalance between the two groups. A two-sided *p* < 0.05 was considered to indicate statistical significance.

Statistical analyses, model construction and evaluations were performed using the Deepwise Multimodal Research Platform version 2.1 (https://keyan.deepwise.com, Beijing Deepwise & League of PHD Technology Co., Ltd, Beijing, China.) and the R software package (version 4.1.2).

## Results

### Clinicopathological characteristics of the investigated patients

Table 1 lists the detailed clinicopathological characteristics of the patients in the training (*n* = 112), test (*n* = 29), and validation (*n* = 40) sets. Among the 181 patients enrolled in this study, 134 (74.0%) were males, who were aged 31 to 86 years. There were 64 pathologic agreement cases, while 117 were pathologic disagreement cases. Under the diagnostic approach of endoscopy, intestinal-type GC was diagnosed in the largest number of patients (84/181), while mixed-type GC was diagnosed under gross pathology in the largest number of patients (72/181).

**Table 1:** Characteristics of patients in the training set, test set, and validation cohort

Characteristic	Training set		*p value*	Test set		*p value*	Validation cohort		*p value*
	Agreement group (n = 69)	Disagreement group (n = 43)		Agreement group (n = 18)	Disagreement group (n = 11)		Agreement group (n = 30)	Disagreement group(n = 10)	
Age	62.449 ± 11.512	56.628 ± 10.137	0.008^*^	61.611 ± 10.617	62.000 ± 9.633	0.922	59.167 ± 9.527	56.700 ± 9.719	0.485
Gender			0.232			1.000			0.693
Men	55 (79.7%)	30 (69.8%)		13 (72.2%)	8 (72.7%)		20 (66.7%)	8 (80.0%)	
Female	14 (20.3%)	13 (30.2%)		5 (27.8%)	3 (27.3%)		10 (33.3%)	2 (20.0%)	
Lauren type			0.404			0.785			0.208
Intestinal type	30 (43.5%)	24 (55.8%)		8 (44.4%)	6 (54.5%)		14 (46.7%)	2 (20.0%)	
Mixed type	21 (30.4%)	9 (20.9%)		4 (22.2%)	3 (27.3%)		9 (30.0%)	6 (60.0%)	
Diffuse type	18 (26.1%)	10 (23.3%)		6 (33.3%)	2 (18.2%)		7 (23.3%)	2 (20.0%)	
Lymph nodes Enlarged			0.208			0.694			0.278
Yes	47 (68.1%)	34 (79.1%)		13 (72.2%)	7 (63.6%)		9 (30.0%)	5 (50.0%)	
No	22 (31.9%)	9 (20.9%)		5 (27.8%)	4 (36.4%)		21 (70.0%)	5 (50.0%)	
Location			0.234			0.622			0.246
Cardia	5 (7.2%)	7 (16.3%)		4 (22.2%)	1 (9.1%)		8 (26.7%)	5 (50.0%)	
Non-cardia	64 (92.8%)	36 (83.7%)		14 (77.8%)	10 (90.9%)		22 (73.3%)	5 (50.0%)	
Thickness (mm)	15.988 ± 5.837	15.941 ± 4.944	0.965	16.910(14.270–22.235) ^a^	15.870(14.190–16.145) ^a^	0.256	16.542 ± 5.036	18.043 ± 7.894	0.486
Longest diameter	51.020(39.400–61.870) ^a^	52.760(39.135–65.245) ^a^	0.735	68.151 ± 32.823	50.374 ± 11.904	0.097	50.537 ± 20.657	49.085 ± 17.577	0.843

Values are the number (percentage) or mean value ± SD or median (interquartile range)

*p < 0.05

^a^Variables were nonnormally distributed. Note—Lauren type determined by endoscopic biopsy. Lymph nodes enlarged, tumour location and size were assessed on CT images

### Radiomics feature selection and radiomics signature building

Of the 854 quantitative features extracted from the CT images, 541 features showed an ICC > 0.75 in the reproducibility analysis. After omitting redundancy using the Pearson’s correlation analysis, 158 features from each patient were used for further selection. By applying the F-test, 48 independent radiomic features were determined as predictive features to build the RS. These features included 13 first-order features, 2 shape features, and 33 texture features, as shown in Additional file 1: Table S2.

**Fig. 2:**
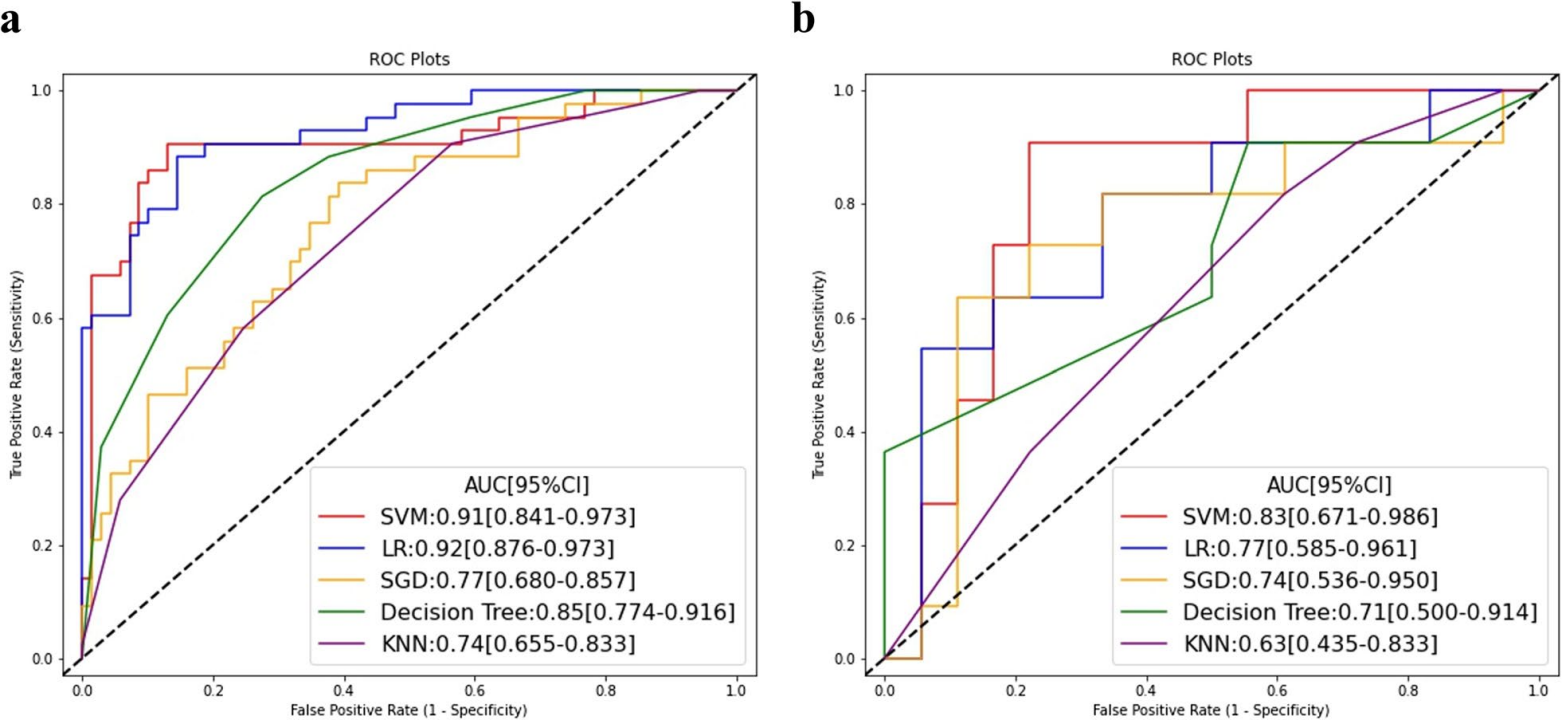
ROC curves for radiomics signatures derived from five machine learning classifiers in (**A**) training set, and (**B**) test set, respectively

Table 2 summarizes the predictive performance of the five RS in the training and test sets. Moreover, the ROC curves of the signatures are shown in Fig. 2.

**Table 2:** Performance of five radiomics signature on the training and test sets

Cohort	AUC [95% CI]	F1 score	Sensitivity	Specificity	NPV	PPV
LR						
Training set	0.92 [0.876–0.973]	0.80	0.77	0.91	0.86	0.85
Test set	0.77 [0.584–0.961]	0.61	0.64	0.72	0.76	0.58
SVM						
Training set	0.91 [0.841–0.973]	0.83	0.81	0.91	0.85	0.89
Test set	0.83 [0.671–0.986]	0.67	0.64	0.83	0.7	0.79
DT						
Training set	0.85 [0.774–0.916]	0.72	0.81	0.72	0.86	0.65
Test set	0.71 [0.499–0.914]	0.52	0.64	0.5	0.69	0.44
SGD						
Training set	0.77 [0.679–0.857]	0.63	0.70	0.67	0.78	0.57
Test set	0.74 [0.536–0.950]	0.67	0.73	0.72	0.81	0.62
KNN						
Training set	0.74 [0.655–0.833]	0.41	0.28	0.94	0.68	0.75
Test set	0.63 [0.435–0.833]	0.27	0.19	0.89	0.64	0.5

*AUC* area under the curve; *CI* confidence interval; *NPV* negative predictive value; *PPV* positive predictive value; *LR* logistic regression; *SVM* support vector machine; *DT* decision tree; *SGD* stochastic gradient descent; *KNN* k-nearest neighbors

### Best radiomics signature evaluation and its DECT generalization testing

A comparison of the discrimination of the five RS is shown in Additional file 1: Table S3. In the test set, only SVM radiomics signature achieved good prediction accuracy (AUC > 0.80). Furthermore, the SVM signature possesses favorable stability between the training and test sets (Delong test: *p* = 0.372); thus, it was selected as the best signature for evaluating disagreement status in the Lauren classification. The optimum cutoff value of the SVM signature determined by ROC curve analysis in the training set was 0.391.

**Fig. 3:**
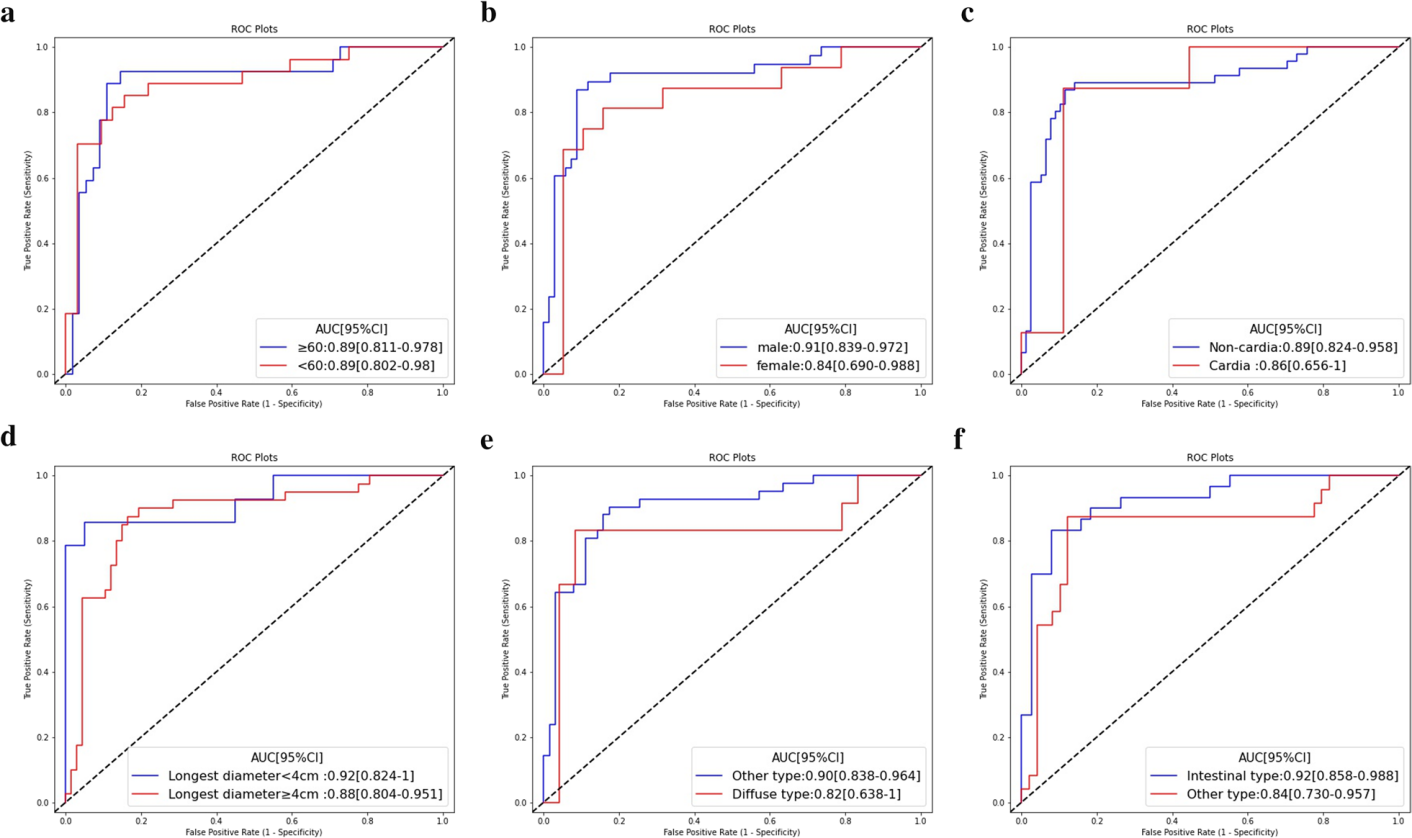
Subgroup analysis of the best radiomics signature (SVM) for all the SECT cohort patients (n = 141). Subgroup analysis were performed on age (**A**), sex (**B**), tumor location (**C**), longest diameter (**D**), and Lauren classification determined by endoscopy (**E**, **F**)

The Mann–Whitney U test showed good correlation between the best RS derived from SVM and pathological disagreement status in the training set (*p* < 0.001) and test set (*p* = 0.03). Moreover, subgroup analysis showed that the performance of the best radiomics imaging biomarker was not affected by age, sex, tumor location, longest diameter, or Lauren classification determined by endoscopy, indicating its independent diagnostic value in different types of GC populations (Fig. 3). Interestingly, the AUCs for distinguishing the pathologic agreement group and disagreement group in the DECT validation cohort were 0.71 (95% CI:0.535–0.875). The Delong test showed a significant difference between the AUC in the training set and validation cohorts (*p* = 0.035), indicating that although the performance of the RS trained on SECT imaging was fair, it still could not be stably generalized to DECT imaging in GC. ROC curves and detailed performances of the best RS in the validation cohort are illustrated in Additional file 1: Fig S1 and Table S4.

### Development and validation of the clinical-radiomics nomogram

**Fig. 4:**
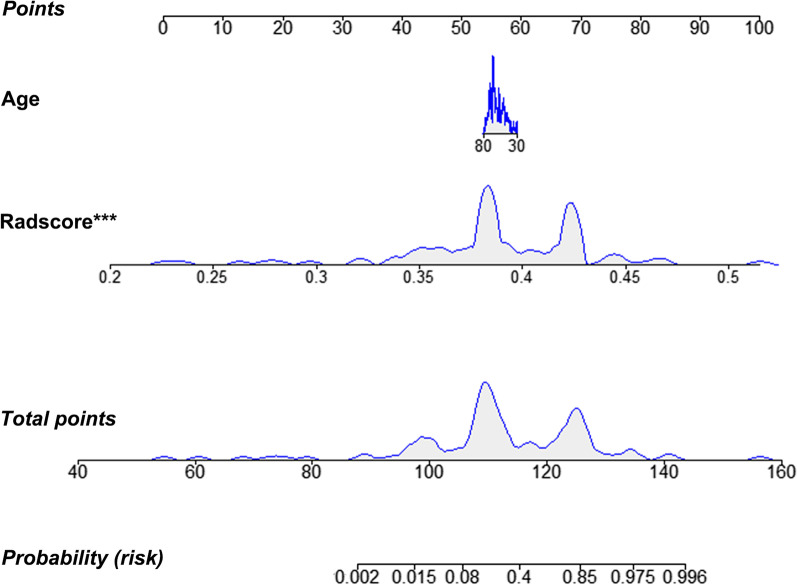
The nomogram based on best radiomic signature and clinical factor

**Fig. 5:**
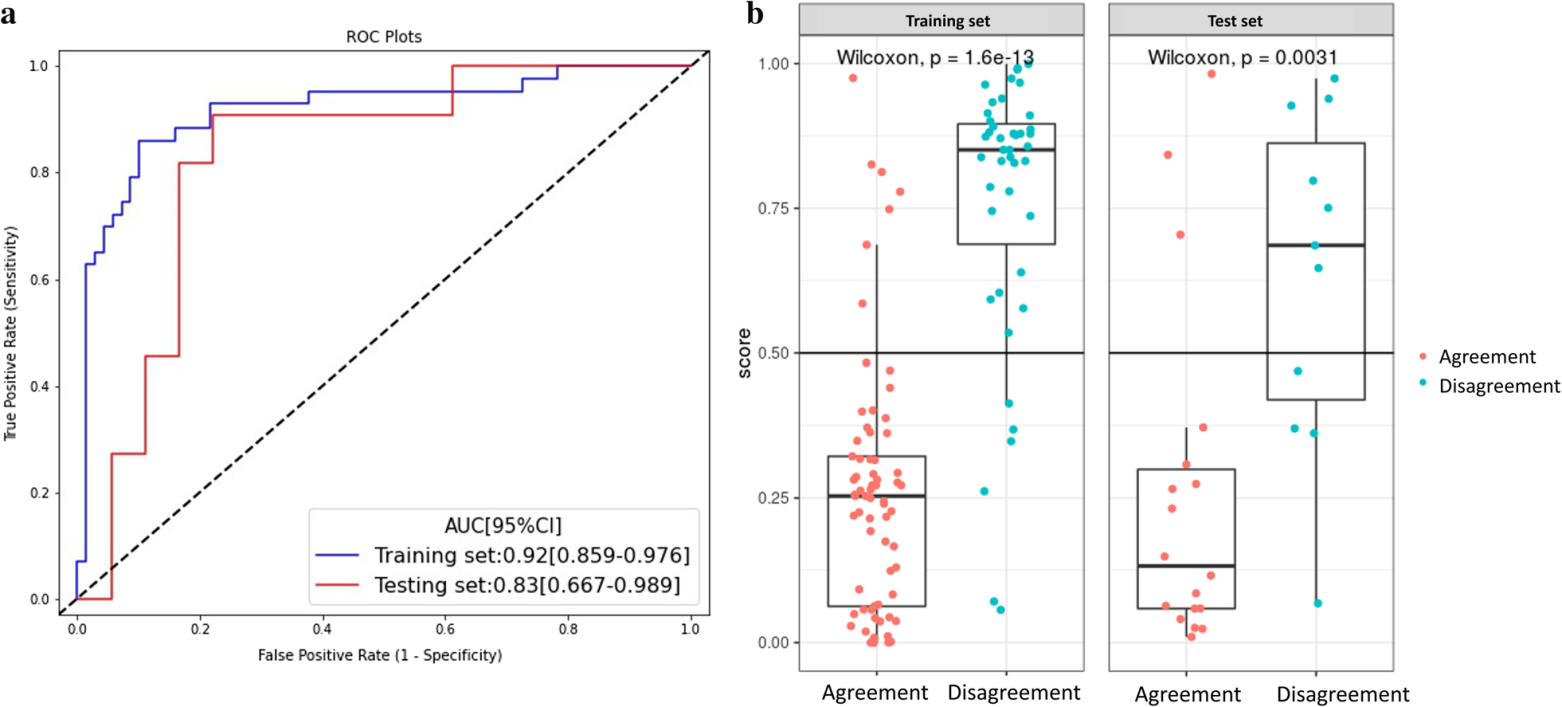
ROC curves of the nomogram in training and test set (**A**), and the boxplots of patients’ nomogram scores between agreement and disagreement groups (**B**)

**Fig. 6:**
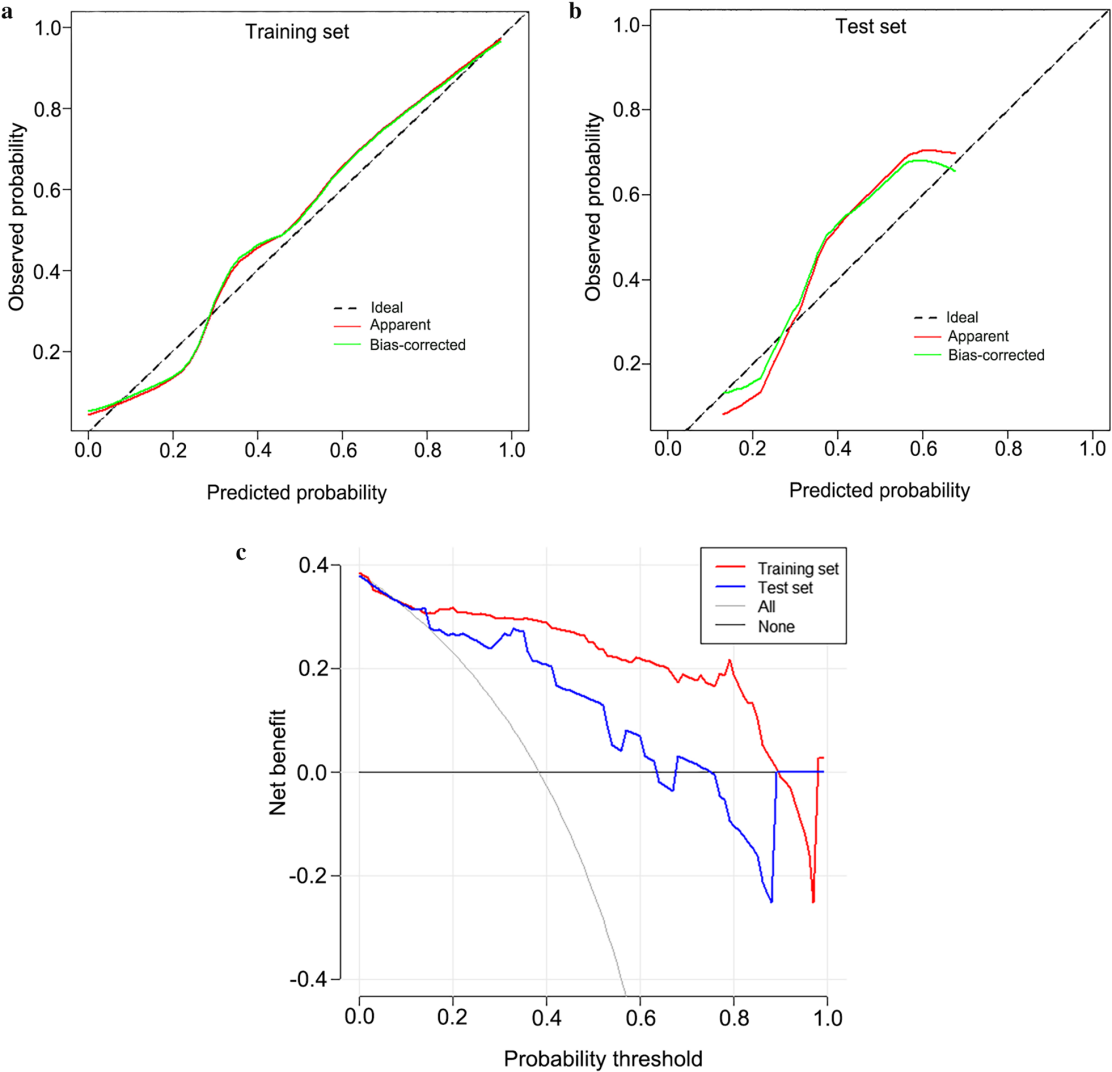
Calibration curves and decision curve analysis of the nomogram. Calibration curves of the nomogram showed good agreement between the predicted and observed pathologic disagreement probability in both the training (**A**) and test set (**B**). The Hosmer–Lemeshow test yielded a nonsignificant statistic (*p*-value = 0.115, and 0.226), suggesting there is no significant departure. Decision curves analysis (**C**) for training set and validation set indicated that when the nomogram is used to predict the risk (probability) of disagreement pathological status, patients could obtain better clinical benefits within wide range of risk (probability) threshold in both sets. The gray curve represents the hypothesis that all pathological status were discordant. The black line represents the hypothesis that no disagreement pathological status

In the training set, the age of the disagreement group was significantly lower than that of the agreement group (*p* < 0.01). Multivariable analysis identified this clinical variable as an independent predictor of pathological disagreement (*p* = 0.01) (Additional file 1: Table S5). A clinical-radiomics nomogram was subsequently developed using age and SVM RS (Fig. 4). Notably, given that the SECT-based SVM signature cannot be stably generalized to the DECT validation cohort, we constructed only the nomogram in the SECT cohort. The proposed nomogram showed powerful discriminative ability, with AUC of 0.92 (95% CI: 0.859–0.976) and 0.83 (95% CI: 0.667–0.989) in the training and test sets, respectively (Fig. 5). The optimum cutoff value determined by ROC curve analysis in the training set was 0.38. The sensitivity, specificity, F1 score, NPV, and PPV are summarized in Table 3. The calibration curves (Fig. 6A, B) and DCA (Fig. 6C) confirmed the good calibration and clinical utility of the nomogram.

**Table 3:** Clinical-radiomics nomogram performance

Cohort	AUC [95% CI]	F1 score	Sensitivity	Specificity	NPV	PPV
Clinical-radiomics nomogram						
Training set	0.92 [0.855–0.976]	0.85	0.86	0.90	0.91	0.84
Test set	0.82 [0.662–0.985]	0.67	0.64	0.83	0.79	0.7

*AUC* area under the curve; *CI* confidence interval; *NPV* negative predictive value; *PPV* positive predictive value

## Discussion

In this observational study, we explored the challenging task of identifying GC patients whose Lauren classification was misdiagnosed via endoscopic biopsy, using a radiomics approach. The purpose was to reduce errors in pathological findings caused by unsuccessful biopsy tissue sampling, providing a potential tool for the preoperative diagnosis of GC. Our proposed nomogram, which incorporates radiomics and clinical signatures, successfully stratified biopsy-misclassified patients in the training and test sets.

Two previous studies compared the Lauren classification in matched biopsy and resection specimens of patients with GC and showed that the overall histological diagnostic disagreement between the two specimens reached 17% (65/382) and 26% (26/100), respectively [17, 18]. However, the current study showed a higher proportion of disagreement classification (35.4% [64/181]). To our knowledge, few studies have explored the clinical risk factors for disagreement in pathology, whereas our study innovatively mined disagreement-associated radiomic imaging biomarkers. In recent years, radiomics has proven to be effective in the characterization of GC pathology, including depth of tumor infiltration assessment [19], determination of the Lauren classification subtype [20], evaluation of the tumor immune microenvironment [21], prediction of lymph node metastasis [22], lymphovascular invasion [23], and HER2 status [24], suggesting that radiomics may hold potential for evaluating the pathologic agreement between the two types of specimens in GC. Therefore, we conducted the present study.

Some scholars have used radiomics features originating from multiple types of CT images to construct radiomics models, including multi-phase images [25], multi-material density images [16], and multi-dimensional images (2D images and 3D images) [26]. However, to our knowledge, few studies have been conducted on the construction of models comprising multiple algorithms based on the same set of radiomics features. Zhang et al. adopted three machine learning classifiers to develop CT-based RS to predict EGFR mutation status in lung adenocarcinoma. They found that an optimal model was developed using an SVM algorithm [27]. Mao et al. compared the performance of five machine learning classifiers in differentiating primary liver cancer from metastatic liver cancer. Their results showed that LR outperformed the other classifiers, with an accuracy of 0.843 ± 0.078 [28]. In this study, although the SVM classifier (linear kernel) was evaluated as the best prediction signature, no significant difference was observed between its performance and that of the LR classifier. This is most likely because our feature selection steps effectively select radiomic features that are more sensitive to current clinical tasks, allowing the linear model to perform well.

An attractive innovation of our study is that, in validating the best RS trained on the SECT cohort, we performed external validation of the signature by including a dataset of dual-energy imaging. Although radiomics has been extensively studied in cancer settings, it is not fully understood whether radiomics can be transferred between dual-energy CT and single-energy CT. Theoretically, single-energy CT images at 120 kV have an image quality equivalent to that of dual-energy VMIs at 70 keV. However, in a phantom study, Chen et al. found that the majority of radiomic features were not reproducible between SECT and DECT [29]. As they did not perform actual clinical task validation, we conducted a pilot study. We found that the radiomic model trained on SECT could not be stably generalized to the DECT cohort. Furthermore, in the clinic, the acquisition and processing time of dual-energy CT images are notably higher than those of single-energy CT images. Thus, from a pragmatic point of view, the generalization of the radiomics model between different CT images is not worth promoting.

Age is closely related to the clinicopathologic and molecular features of GC [30]. Lee et al. revealed that age, sex, tumor size, and tumor location were risk factors for pathological discordance in patients with GC [7]. Although the current study only found that age was significant in the univariate analysis (*p* < 0.05), we also attempted to include the other clinical factors mentioned above in the regression analysis. The final result suggested that age was the only clinical predictor of pathological disagreement. A slight improvement in predictive power was observed when age, a more easy-to-obtain clinical variable, was added to the best RS. We speculate that the slight association between clinicopathological features and pathologic disagreement in the present study could be because of the stronger correlation between intratumoral heterogeneity and pathologic disagreement. Consequently, intratumoral heterogeneity associated with disagreement could be reflected, to a large extent, by the beyond-visual radiomics features extracted from the CT images.

The present study has some limitations. First, because of the retrospective nature of the training and test sets, different scanners were used in the SECT dataset, which may result in some inherent bias. Future studies need to focus on the standardization of radiomics to obtain high-quality data. Second, tumor segmentation in our study was performed on the largest slice of 2D CT images, which might not be sufficiently representative of the whole tumor. However, some scholars have demonstrated that 2D segmentation can avoid more image noise and the interference of effective information, and even 2D radiomics model predictive performance in GC is not inferior to that of 3D radiomics model [19, 31]. Third, this was a single-center study with an insufficient sample size; therefore, the heterogeneity of GC among different regional populations was not considered. We have an ongoing collaboration with other centers to recruit large samples of patients with GC. Additionally, in the comparison of the models, we only analyzed them in terms of predictive performance but neglected to pay attention to how time-consuming it is to develop the models.

In conclusion, we developed a clinical-radiomics nomogram that allows noninvasive evaluation of pathological disagreement status in GC. Such imaging biomarkers may hold promise as transitional tools between endoscopic biopsy and gross pathology. Moreover, the proposed SECT cohort-based radiomics model obtained with SVM classifier could not be stably generalized to DECT cohort.

## Supplementary Information

**Additional file 1.** **Appendix E1**: Study population. **Appendix E2**: Image acquisition and reconstruction parameters. **Appendix E3**: Tumor segmentation and extraction of radiomic features. **Supplementary Table S1**: Imaging Scheme and Acquisition Parameters. **Supplementary Table S2**: Final retained 48 independent radiomics features. **Supplementary Table S3.1**: Comparison of discrimination (AUC value) of five models in training set. **Supplementary Table S3.2**: Comparison of discrimination (AUC value) of five models in test set. **Supplementary Table S4**: Detailed Performance of the SVM model in the DECT validation cohort. **Supplementary Table S5**: Multivariate Logistic Regression Analysis for Significant Clinical Variable. **Supplementary Figure S1**. ROC curves of the SVM model in the DECT validation cohort.

## Data Availability

The datasets used and/or analyzed during the current study are available from the corresponding author on reasonable request.

## Abbreviations

AUC: Area under the receiver operating characteristic curve
DCA: Decision curve analysis
DECT: Dual-energy computed tomography
DT: Decision tree
GC: Gastric cancer
KNN: K-Nearest Neighbors
LR: Logistic regression
NPV: Negative predictive value
PPV: Positive predictive value
ROC: Receiver operating characteristic
RS: Radiomics signatures
SECT: Single-energy computed tomography
SGD: Stochastic gradient descent
SVM: Support vector machine
VMIs: Virtual monochromatic images
