# Supplementary material for: Virtual biopsy using CT radiomics for evaluation of disagreement in pathology between endoscopic biopsy and postoperative specimens in patients with gastric cancer: a dual-energy CT generalizability study
Source: Insights Imaging. 2023 Jul 5;14:118. doi: 10.1186/s13244-023-01459-w (PMC10323070; doi:10.1186/s13244-023-01459-w)
Supplement: Supplementary file 1 — Additional file 1. Appendix E1: Study population. Appendix E2: Image acquisition and reconstruction parameters. Appendix E3: Tumor segmentation and extraction of radiomic features. Supplementary Table S1: Imaging Scheme and Acquisition Parameters. Supplementary Table S2: Final retained 48 independent radiomics features. Supplementary Table S3.1: Comparison of discrimination (AUC value) of five models in training set. Supplementary Table S3.2: Comparison of discrimination (AUC value) of five models in test set. Supplementary Table S4: Detailed Performance of the SVM model in the DECT validation cohort. Supplementary Table S5: Multivariate Logistic Regression Analysis for Significant Clinical Variable. Supplementary Figure S1. ROC curves of the SVM model in the DECT validation cohort. [file 13244_2023_1459_MOESM1_ESM.docx]

**Virtual biopsy using CT radiomics for evaluation of disagreement in pathology**

**between endoscopic biopsy and postoperative specimens in patients with**

**gastric cancer: A dual–energy CT generalizability study**

***ELECTRONIC SUPPLEMENTARY MATERIAL***

**List of Supplementary Materials:**

**Appendix E1:** Study population

**Appendix E2:** Image acquisition and reconstruction parameters

**Appendix E3:** Tumor segmentation and extraction of radiomic features

**Supplementary Table S1：**Imaging Scheme and Acquisition Parameters

**Supplementary Table S2：**Final retained 48 independent radiomics features

**Supplementary Table S3.1:** Comparison of discrimination (AUC value) of five models in training set

**Supplementary Table S3.2:** Comparison of discrimination (AUC value) of five models in test set

**Supplementary Table S4:** Detailed Performance of the SVM model in the DECT validation cohort.

**Supplementary Table S5:** Multivariate Logistic Regression Analysis for Significant Clinical Variable.

**Supplementary FigureS1**

**Appendix E1: Study population**

The main inclusion criteria were (1) pathologically confirmed gastric adenocarcinoma; (2) diagnosis of nonmetastatic locally advanced GC (cT2-4aNxM0) according to the 8th Edition American Joint Committee on Cancer (AJCC) tumor-node-metastasis (TNM) staging criteria; (3) underwent baseline abdominal contrast-enhanced CT; (4) underwent radical gastrectomy; and (5) both biopsy and gross pathology findings were available. The main exclusion criteria were (1) diagnosis of other gastric malignant neoplasms; (2) previous anticancer therapy (e.g., radiotherapy, chemotherapy, or targeted therapy); (3) poor CT imaging quality to perform measurements (e.g., severe artifacts); and (4) incomplete clinicopathological data.

**Appendix E2: Image acquisition and reconstruction parameters**

**Details on imaging protocol**

The patients fasted for 6-8 h and were then given 800-1000 ml of warm water 20 min before the examination to ensure good gastric fullness. Besides that, the patients drank 150-200ml of warm water immediately before performing the examination, as these patients needed to focus on the stomach and duodenum. The CT examinations were performed while holding the breath, with the patient in the supine position or, in exceptional cases (eg. the gastric sinus and pylorus lesions), in the prone or lateral position. Plain CT images were acquired first, followed by dual-phase (arterial and venous) enhanced CT images after a high-pressure syringe was used to inject non-ionic contrast agent through the anterior elbow vein. Arterial phase (AP) and venous phase (VP) CT images were acquired 30 s and 70 s after postinjection delays, respectively. All SECT and DECT images were reconstructed using a standard kernel with a slice thickness of 5 mm. Acquisition parameters are summarized in **TableS1**.

**Appendix E3: Tumor segmentation and extraction of radiomic features**

The region of interest (ROI) was delineated around the outline of the tumor on the axial slice with the largest cross-sectional tumor area by a radiologist blinded to the pathological and clinical outcomes. One month later, the radiologist randomly selected 30 CT images for ROI re-segmentation to evaluate intra-observer reproducibility.

The radiomic features extracted from the software (first-order, shape, and texture) were computed using “PyRadiomics” (version 2.2.0), in accordance with the benchmarks of Image Biomarker Standardization Initiative. In addition to the features from the original images, some features were extracted from the image via preprocessing with wavelet filtering.

**Supplementary TableS1.** Imaging Scheme and Acquisition Parameters

| **Parameters** | **SECT** | **DECT** |
| --- | --- | --- |
| CT scanner | Discovery CT (GE Healthcare, USA);  Brilliance iCT (Philips Health-care System, Netherlands) | Revolution Apex (GE Healthcare, USA) |
| Tube voltage | 120 kVp | rapid tube voltage switching between the 80- and 140-kVp |
| Tube Current | 290-650mAs | 405 mA |
| Rotation time | 0.5s | 0.6s |
| Contrast agent | iohexol [Omnipaque, GE Healthcare] | iohexol [Omnipaque, GE Healthcare] |
| *concentration* | 350 mgI/ml | 350 mgI/ml |
| *dosage* | 1.2-1.3 ml/kg | 1.2-1.3 ml/kg |
| *flow rate* | 2.0-4.0 ml/s | 2.0-4.0 ml/s |
| FOV | 500×500 mm | 400mm×400mm |
| Reconstructed image/Section thickness | 120kVp/ 5mm | 70keV VMI/ 5mm |

Note——DECT = Dual-energy computed tomography; SECT = Single-energy computed tomography; VMI = Virtual monochromatic images.

**Supplementary TableS2.** Final retained 48 independent radiomics features

| wavelet-LLL_glcm_ClusterShade  wavelet-LLL_glcm_Contrast  wavelet-HLH_firstorder_Median  wavelet-HLH_gldm_DependenceVariance  wavelet-LHL_glcm_Imc1  wavelet-HLH_gldm_LargeDependenceLowGrayLevelEmphasis  wavelet-HHH_firstorder_Median  original_glcm_ClusterShade  wavelet-HLH_gldm_LargeDependenceEmphasis  wavelet-LHL_glszm_SizeZoneNonUniformityNormalized  wavelet-HLL_glcm_Idn  wavelet-HLL_firstorder_10Percentile  wavelet-HHL_firstorder_Median  wavelet-LHH_glrlm_ShortRunLowGrayLevelEmphasis  wavelet-LLL_glcm_DifferenceAverage  wavelet-LHH_gldm_SmallDependenceLowGrayLevelEmphasis  wavelet-LLH_firstorder_TotalEnergy  wavelet-LHH_ngtdm_Busyness  wavelet-HHL_glszm_LargeAreaHighGrayLevelEmphasis  wavelet-LLL_ngtdm_Contrast  wavelet-LLH_firstorder_Maximum  wavelet-HHH_gldm_LargeDependenceHighGrayLevelEmphasis  wavelet-LHL_firstorder_MeanAbsoluteDeviation  original_shape_SurfaceVolumeRatio  wavelet-HHH_firstorder_Mean  wavelet-LLL_glcm_Imc1  wavelet-HLL_ngtdm_Busyness  wavelet-HLH_glszm_ZoneEntropy  wavelet-HLH_glcm_DifferenceEntropy  wavelet-HHL_glrlm_RunEntropy  original_glcm_DifferenceVariance  wavelet-LLL_glcm_Imc2  original_shape_Maximum2DDiameterColumn  original_shape_VoxelVolume  wavelet-LLH_glszm_LargeAreaHighGrayLevelEmphasis  wavelet-LHH_gldm_DependenceEntropy  original_glcm_Idn  wavelet-LLL_glcm_Idmn  wavelet-LLH_glrlm_GrayLevelNonUniformity  wavelet-LLL_glcm_JointEnergy  wavelet-LLL_gldm_DependenceVariance  wavelet-HHH_firstorder_Variance  wavelet-HHH_glcm_JointEntropy  wavelet-HHL_firstorder_Uniformity  original_glcm_Idmn  original_shape_MajorAxisLength  original_glszm_ZonePercentage  wavelet-LHH_gldm_DependenceNonUniformityNormalized |  |
| --- | --- |

**Supplementary Table S3.1** Comparison of discrimination (AUC value) of five models in training set

| **LR** |  |  |  |  |
| --- | --- | --- | --- | --- |
| **SVM** | 0.289 |  |  |  |
| **SGD** | 0.000 ^**^ | 0.001 ^**^ |  |  |
| **DT** | 0.021 ^*^ | 0.133 | 0.141 |  |
| **KNN** | 0.001 ^**^ | 0.005 ^**^ | 0.725 | 0.065 |
|  | **LR** | **SVM** | **SGD** | **DT** |

Note—— ^*^p < 0.05, ^**^p < 0.01. LR: Logistic Regression, SVM: Support Vector Machine, DT: Decision Tree, SGD: Stochastic Gradient Descent, and KNN: K Nearest Neighbors.

**Supplementary Table S3.2** Comparison of discrimination (AUC value) of five models in test set

| **LR** |  |  |  |  |
| --- | --- | --- | --- | --- |
| **SVM** | 0.306 |  |  |  |
| **SGD** | 0.780 | 0.382 |  |  |
| **DT** | 0.574 | 0.333 | 0.811 |  |
| **KNN** | 0.225 | 0.088 | 0.328 | 0.556 |
|  | **LR** | **SVM** | **SGD** | **DT** |

Note—— LR: Logistic Regression, SVM: Support Vector Machine, DT: Decision Tree, SGD: Stochastic Gradient Descent, and KNN: K Nearest Neighbors.

**Supplementary Table S4.** Detailed Performance of the SVM model in the DECT validation cohort.

|  | **AUC** | **F1 Score** | **Sensitivity** | **Specificity** | **NPV** | **PPV** |
| --- | --- | --- | --- | --- | --- | --- |
| **SVM** | 0.705 | 0.51 | 1 | 0.37 | 1 | 0.34 |

Note—— SVM: Support Vector Machine; NPV: Negative Predictive Value; PPV: Positive Predictive Value.

**Supplementary Table S5.** Multivariate Logistic Regression Analysis for Significant Clinical Variable.

| **Variable** | **odds ratio value** | **95%CI** | ***p*** |
| --- | --- | --- | --- |
| Age^a^ | 0.954 | 0.921-0.989 | 0.01 |

^a^ Per increase of 1 unit (year).


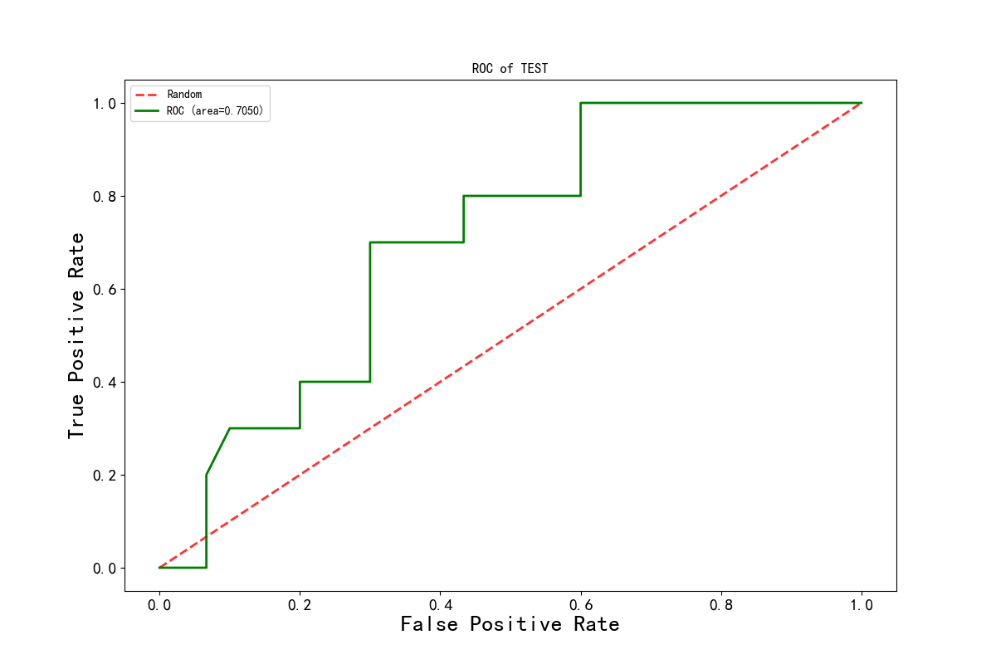


**Supplementary FigureS1.** ROC curves of the SVM model in the DECT validation cohort.

Note——ROC=Receiver operating characteristic. SVM= Support Vector Machine. DECT: Dual-energy computed tomography.
